# Self-synchronization of reinjected droplets for high-efficiency droplet pairing and merging

**DOI:** 10.1038/s41378-023-00502-6

**Published:** 2023-03-09

**Authors:** Lang Nan, Tianjiao Mao, Ho Cheung Shum

**Affiliations:** 1 Advanced Biomedical Instrumentation Centre, Hong Kong Science Park, Shatin, New Territories, Hong Kong China; 2 Department of Mechanical Engineering, The University of Hong Kong, Pokfulam Road, Hong Kong, China

**Keywords:** Engineering, Chemistry

## Abstract

Droplet merging serves as a powerful tool to add reagents to moving droplets for biological and chemical reactions. However, unsynchronized droplet pairing impedes high-efficiency merging. Here, we develop a microfluidic design for the self-synchronization of reinjected droplets. A periodic increase in the hydrodynamic resistance caused by droplet blocking a T-junction enables automatic pairing of droplets. After inducing spacing, the paired droplets merge downstream under an electric field. The blockage-based design can achieve a 100% synchronization efficiency even when the mismatch rate of droplet frequencies reaches 10%. Over 98% of the droplets can still be synchronized at nonuniform droplet sizes and fluctuating reinjection flow rates. Moreover, the droplet pairing ratio can be adjusted flexibly for on-demand sample addition. Using this system, we merge two groups of droplets encapsulating enzyme/substrate, demonstrating its capacity to conduct multi-step reactions. We also combine droplet sorting and merging to coencapsulate single cells and single beads, providing a basis for high-efficiency single-cell sequencing. We expect that this system can be integrated with other droplet manipulation systems for a broad range of chemical and biological applications.

## Introduction

Droplet microfluidics enables generation of uniformly sized droplets as reaction vessels to perform chemical and biological assays in a stable and controllable manner^1,2^. To conduct multi-step reactions, several batches of samples need to be added sequentially^3,4^. Thus, multiple sets of droplets carrying different reagents or targets should be paired and merged sequentially^5–7^. This requires precise synchronization of each set of droplets with very similar frequencies^8,9^. However, microfluidic droplets tend to be unstable after rounds of processing, especially when long-term incubation and heating are involved^10^. These processing steps frequently induce droplet coalescence and lead to non-uniformly sized droplets, thus changing the reinjection frequency and disrupting droplet merging^11^. Moreover, fluctuations in flow rates could also generate reinjection errors and aggravate the instability of droplet synchronization and merging^12,13^. As a result, even when merging two sets of droplets with uniform size, 15% of them are incorrectly ordered; the error rate further increases when the two sets of droplets have size variations^14^.

Several methods have been developed to enhance the synchronization of in situ generated droplets. For instance, two sets of droplets can be alternately generated through hydrodynamic coupling of two T-junction generation nozzles^15^. When droplets are generated in one of the nozzles, the junction is transiently blocked by the aqueous stream, inhibiting the flow of oil coming from the central channel. As a result, the flow rate of oil in the other nozzle increases, and droplet formation is stopped. The alternate blockage of the two junctions leads to stable droplet pairing at various flow rates. A railroad-like channel can also be used to synchronize two trains of droplets generated upstream^16^. The crossflow of carrier oil through the ladder network automatically balances the pressure difference between two droplet flows, thus achieving synchronization efficiencies up to 95%^17^. In addition, some active structures, such as trapping wells^18^ and chambers^19^, have also been developed to synchronize the droplets. Two sets of droplets are immobilized within the structures successively, thus eliminating the mismatch of their arrival times. However, these strategies are only suitable for droplets generated on chip but cannot be applied to reinjected droplets for multi-step reactions.

To further synchronize one set of reinjected droplets with another set of generated droplets, a droplet cleaving strategy is developed^20^. Each reinjected droplet cleaves an aqueous stream into a new droplet, thus generating pairs of rejected and new droplets. Nevertheless, although liquid reagents can be added via on-chip generated droplets, the addition of discrete targets, such as microbeads and cells, to reinjected droplets has low efficiency due to Poisson fluctuation. The most efficient way to beat Poisson statistics and combine discrete targets without sample loss is merging the droplets containing desired targets sorted in a previous step^21^. Nevertheless, to date, established platforms can seldom realize the synchronization and merging of two sets of reinjected droplets.

To address this, we develop a simple yet versatile hydrodynamic resistance-regulated microfluidic device, where automatic droplet synchronization, spacing, and merging are conducted sequentially. The basic synchronization principle is as follows: As one droplet enters the junction, the local hydrodynamic resistance increases significantly, thus transiently blocking subsequent droplets entering from the other channel; once the droplet passes through, the hydrodynamic resistance returns to normal, and the subsequent droplet immediately follows and enters the junction. The periodical blockage in the T-junction results in alternate arrangement of the two sets of droplets, followed by downstream spacing and merging. Leveraging this strategy, we can achieve a synchronization efficiency of 100% for droplets with frequencies mismatched within 10%. Even when the droplet size and reinjection flow rate fluctuate, the synchronization ratio can remain over 98%. Moreover, our strategy is not only accessible to one-to-one droplet pairing but can also be applied to one-to-two and one-to-three droplet matching. In addition, an extra set of droplets can be added and synchronized with previous droplet pairs, providing higher flexibility in sample permutation and combination. To demonstrate the potential of our system to conduct multi-step reactions, we merge two batches of droplets containing two groups of enzymes/substrates and obtained monodisperse droplets with uniform size and homogeneous products of enzymatic cascade reaction. We also use this system to merge two batches of droplets encapsulating single cells and single beads, significantly increasing the efficiency of barcoding. Overall, our microfluidic design enables self-synchronization of 100% of reinjected droplets and opens up new avenues in multi-step chemical and biological reactions.

## Results and discussion

### Workflow of droplet merging

**Fig. 1:**
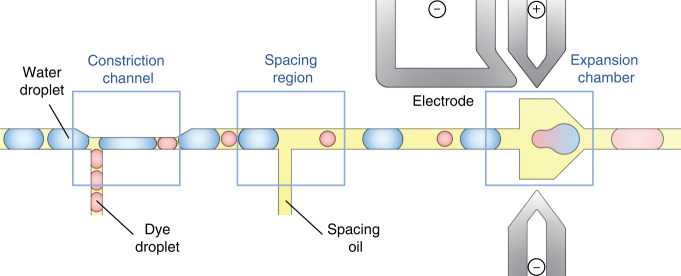
Schematic of the microfluidic design for droplet synchronization, spacing, and merging. Two sets of droplets alternately block each other and enter the channel, followed by spacing and coalescence of the droplet pairs

**Fig. 2:**
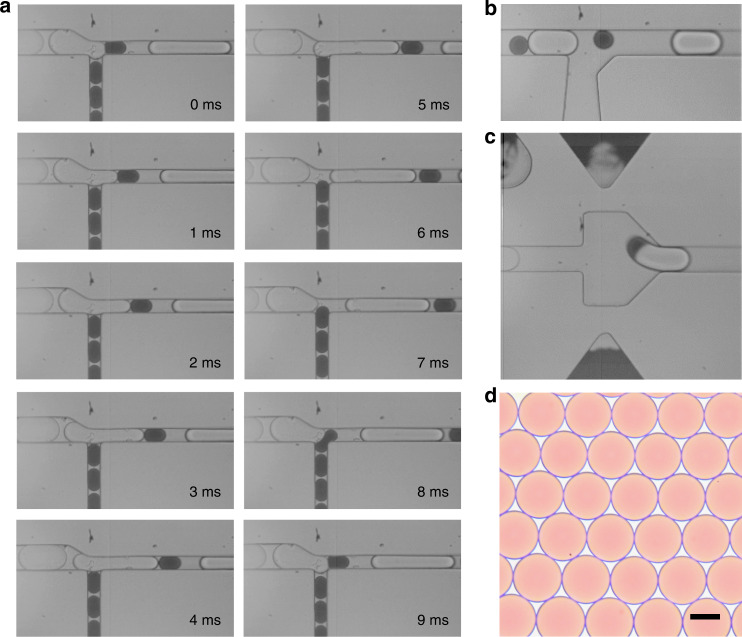
Synchronization, spacing, and merging of two sets of reinjected droplets. **a** Frame-by-frame micrographs showing the process of droplet synchronization in the constriction channel. **b** The synchronized droplets are spaced by the oil flow introduced from the T-junction. **c** Pairs of droplets are in close contact in the expansion chamber and coalesce under the electric field. **d** Optical micrograph showing merged droplets with uniform size and color distribution. Scale bar: 50 μm

The droplet-merging channel consists of three parts: the constriction channel for droplet synchronization, the spacing region for droplet separation, and the expansion chamber for droplet coalescence (Fig. 1, Fig. S1 and Movie. S1). Two sets of droplets with diameters of 35 and 65 μm are reinjected into two separate channels at flow rates of 25 and 90 μL/h, respectively. When one large droplet flows through and blocks the T-shaped junction, a significant increase in the local hydrodynamic resistance is generated. This exerts pressure on the front curved surface of the small droplet, thus balancing the pressure exerted on the back curved surface by the continuous flow. As a result, the small droplet is temporarily immobilized for 0~5 ms. After the large droplet passes through, the lower downstream pressure is restored, causing the small droplet to immediately follow the large droplet and enter the channel (6~7 ms). Likewise, the small droplet transiently blocks the junction and stops the flow of the large droplet (8~9 ms). Then, immediately after the small droplet passes through, the next large droplet enters, starting the next cycle of droplet arrangement (Fig. 2a). In this fashion, the two sets of droplets can enter the channel alternately at a frequency of approximately 111 Hz.

Upon synchronization, the pairs of droplets flow through another T-junction, where an oil flow is introduced from a vertical branch channel to increase the gap between adjacent droplets (Fig. 2b). Although the paired large and small droplets are spaced by the oil, due to their difference in droplet size and velocity, the small droplet can gradually catch up with the large droplet downstream. As the pairs of droplets are guided into the expansion chamber, the velocities of both droplets significantly decrease, causing them to contact one another. Three electrodes are fabricated on both sides of the chamber: The top left one serves as the shield electrode to prevent droplet coalescence during reinjection and arrangement, while the top right and bottom electrodes are used to activate electric field in the chamber. When a voltage is applied, the water-oil interface stabilized by the surfactant is perturbed, triggering droplet coalescence (Fig. 2c). To ensure stable coalescence, the applied voltage should be within the range of 600 Vp-p to 1200 Vp-p. With an insufficient voltage, droplet coalescence is not triggered; excessively high voltages might cause droplet wetting, leading to cross-contamination (Fig. S2). The merged droplets are uniform in size and color distribution, demonstrating the high efficiency of droplet synchronization, spacing, and merging (Fig. 2d).

### Characterization of the synchronization effect

In conventional designs without any enhancement of channel blockage, the synchronization efficiency depends on the matching degree of the large- and small-droplet reinjection frequencies, *f*_1_ and *f*_2_, respectively. During a certain period, *T*, the number of large droplets that have entered the T-junction is given by *n*_1_ = *f*_1_*T*, and the number of small droplets is given by *n*_2_ = *f*_2_*T*. The ratio of one-to-one droplet pairing can thus be expressed as:

1 $$P\,{{{\mathrm{ = 1}}}\,} - \frac{{|n_1 - n_2|}}{{n_1}}\,{{{\mathrm{ = 1}}\,}} - \frac{{|f_1 - f_2|}}{{f_1}}$$

In this manner, even a minor mismatch of the reinjection frequencies can result in synchronization errors and lower the one-to-one pairing ratio. In comparison, our design allows automatic tuning of mismatched frequencies: When the small droplet arrives more frequently than the large droplet, the large droplet temporarily stops the flow of the small droplet and decreases its arrival frequency, and vice versa. Self-synchronization of reinjected droplets and higher merging efficiency can thus be achieved.

**Fig. 3:**
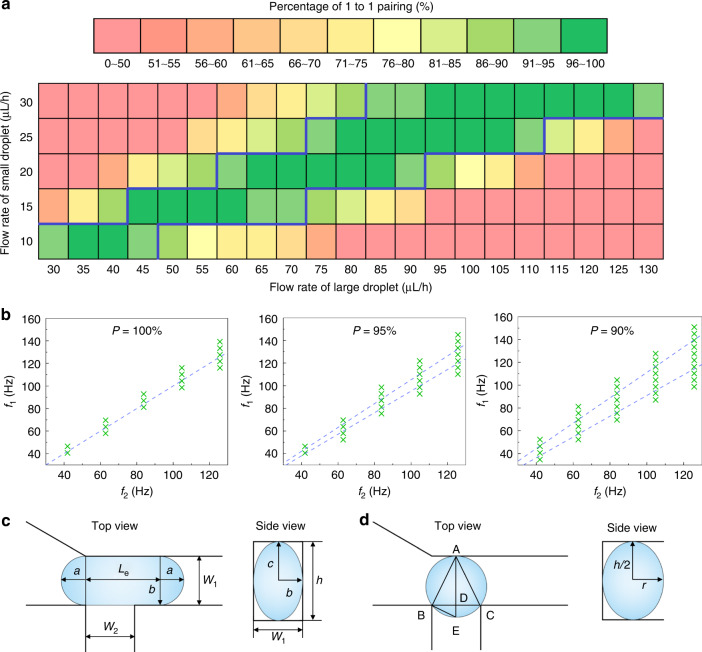
Characterization of the synchronization effect. **a** Heatmap depicting the percentage of one-to-one pairing droplets at various reinjection flow rates. The flow rate of the small droplets ranges from 10 to 30 μL/h at an interval of 5 μL/h, while the large-droplet flow rate ranges from 30 to 130 μL/h. The tested region with a synchronization ratio higher than 90% is outlined by an indigo line. **b** Graphs showing the matched frequencies that can achieve synchronization ratios of 100, 95, and 90%. The cross dots denote the matched frequencies for our device, while the dashed lines indicate the theoretical working ranges of frequencies for the negative control. **c** Top and side views showing the shape of the large droplet entering the constriction channel. **d** Top and side views showing the shape of the small droplet at its critical volume to block the junction

To quantify the synchronization efficiency of our device, we measure the ratio of one-to-one droplet pairing at various reinjection flow rates. The synchronization ratio remains high (over 90%) across a wide range of flow rates, as outlined in Fig. 3a. Although the tested largest flow rates for small and large droplets are 30 μL/h and 130 μL/h, the two droplets can be likewise synchronized when the flow rates further increase until a breakup occurs. In case of a droplet breakup, the droplet would be split into two smaller droplets and could not block the subsequent droplet. For our adopted channel geometries and droplet sizes, the upper thresholds of flow rates for the small and large droplets are 80 and 400 μL/h, respectively (Fig. S3). Moreover, for the five tested flow rates of the small droplets, two to five tested large-droplet flow rates can achieve 100% synchronization efficiency. To further characterize the synchronization efficiency at different sets of reinjection frequencies, we measure the droplet reinjection frequencies at various flow rates, which shows a linear relation for both the large and small droplets (Fig. S4). The flow rates in Fig. 3a can thus be transferred into droplet frequencies to indicate the tolerance in mismatching of frequencies for high-efficiency synchronization (Fig. 3b). The results show that for our device, 100% of droplets can be synchronized when *f*_1_ and *f*_2_ are within 10% of bias (|*f*_1_−*f*_2_ | */f*_1_ < 10%), whereas for the negative control without any enhancement of blockage, *f*_1_ and *f*_2_ must be strictly equal according to Eq. (1). Moreover, for synchronization efficiencies of 95% and 90%, our device also allows wider ranges of mismatching (15% and 20%) than the negative control (5% and 10%).

It should be noted that to enable self-synchronization, the droplet must be able to block the junction and stop the flow of its counterpart. Thus, the droplet size should be matched with the channel geometry within specific ranges to work properly. Specifically, a large droplet enters the junction as a quasielliptical cylinder, which is composed of an elliptical cylinder and two identical semi-ellipsoids, as shown in Fig. 3c. To block the small droplet, the length of the elliptical cylinder (top view), *L*_e_, should be larger than the width of the small droplet channel, *w*_2_. Thus, the minimum volume of the large droplet can be expressed as:

2 $$V_{1,min} = V_e + 2V_s = \pi bcw_2 + 4/3\pi abc$$

Herein, *V*_e_ and *V*_s_ represent the volume of the elliptical cylinder and semi-ellipsoid, respectively; *c* = *h*/2, where *h* is the channel height; and *a* = *b* = *w*_1_/2, where *w*_1_ refers to the width of the constriction channel, since the two semi-ellipsoid can be combined and viewed as a prolate spheroid with the length of two minor axes equal to half of the channel width^22^. For our device, where *w*_1_ = 25 μm, *w*_2_ = 25 μm, and *h* = 40 μm, the minimum volume of the large droplet is calculated to be 33 pL, corresponding to a diameter of approximately 40 μm, which is consistent with our experimental results (Fig. S5a). The small droplet enters the junction as an ellipsoid. At the critical condition when the small droplet can just block the junction, the top view of the small droplet emerges as a circle at the T-junction (Fig. 3d). Thus, the radius of the circle can be described as:

3 $$r\,=\,(l_{\mathrm{AD}}\,+\,l_{\mathrm{DE}})/2\,=\,(l_{\mathrm{AD}}\,+\,{l_{\mathrm{BD}}}^{2}/l_{\mathrm{AD}})/2\,=\,w_{1}/2\,+\,{w_{2}}^{2}/(8w_{1})$$

For our device, the critical volume of the small droplet is calculated to be *V*_2, min_ = 2π*r*^2^*h*/3 = 21 pL. This volume corresponds to a diameter of approximately 34 μm, which is also consistent with our experimental results (Fig. S5b). Based upon the above results, we select 65 and 35 μm as the diameters of the large and small droplets, respectively, for subsequent tests. Despite using these specific sizes of droplets for a demonstration, other sizes of droplets within the working ranges can also be synchronized with a high efficiency by regulating the flow rates. As shown in Table 1, for all the tested groups, 100% synchronization efficiency can be achieved across a wide range of large-droplet flow rates.

**Table 1:** Flow rate of large droplets that can maintain 100% synchronization efficiency for different size combinations

Small droplet size	Large droplet size		
	60 μm	70 μm	80 μm
35 μm	80~100 μL/h	125~145 μL/h	185~210 μL/h
40 μm	60~80 μL/h	95~115 μL/h	135~160 μL/h
45 μm	50~70 μL/h	70~90 μL/h	95~120 μL/h

The flow rate of the small droplet is kept constant at 30 μL/h

### Droplet synchronization at fluctuating droplet sizes and reinjection flow rates

Because the droplet reinjection rate is proportional to the flow rate and inversely proportional to the droplet volume, non-uniformity in droplet size and fluctuations in flow rates are the two most common issues that may change the droplet frequencies^23–25^. In conventional designs, these fluctuations tend to generate trains of unpaired or one-to-multiple paired droplets and lower the synchronization efficiency^26^; in comparison, our device, which can synchronize the frequencies of large and small droplets in case of mismatches, is well suited to overcome the fluctuations and prevent synchronization errors. To demonstrate its superior synchronization effect, we test the efficiency of droplet pairing under fluctuating reinjection conditions, including mixing droplets with different sizes and varying the flow rates periodically, and compare the performance of our device with that of a negative-control device. The dimensions of the junction are set as *w*_*1*_ = 75 μm and *w*_*2*_ = 40 μm for the negative-control device. According to Eqs. (2) and (3), the tested droplet diameters of 35 and 65 μm are below the thresholds (74 and 80 μm) to block the junction, thus presenting no synchronization effect (Fig. S6).

**Fig. 4:**
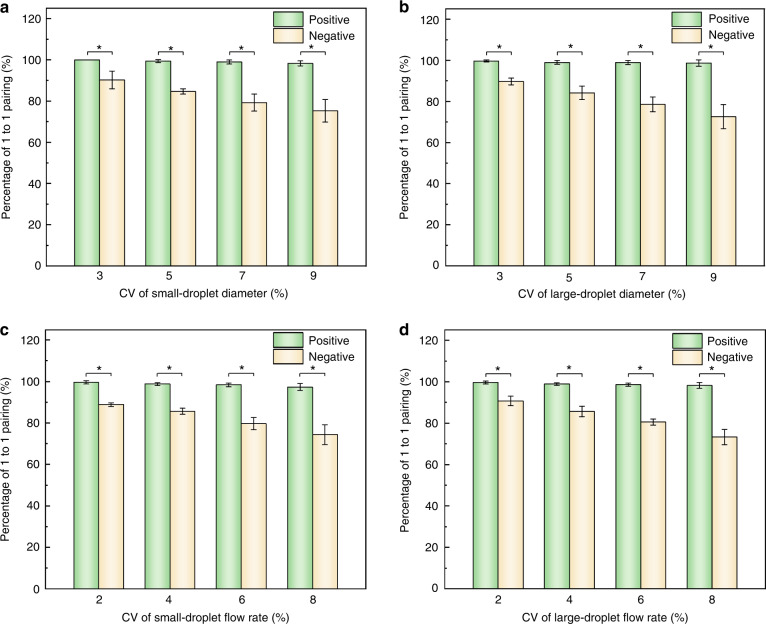
Synchronization efficiencies with fluctuating droplet sizes and reinjection flow rates. Histograms showing the percentage of 1 to 1 pairing droplets at nonuniform diameters of **a** small droplets and **b** large droplets with CVs ranging from 3 to 9% and at fluctuating flow rates of **c** small droplets and **d** large droplets with CVs ranging from 2 to 8%

To demonstrate the ability of our design to accommodate size variation, we first generate four groups of small droplets with the coefficient of variation (CV) of diameter ranging from 3 to 9% (Fig. S7a), followed by measuring the percentage of one-to-one droplet pairing for each CV. For our device, the synchronization ratio is maintained at over 98% for all the tested CVs, whereas for the negative device, the synchronization ratio decreases significantly with increasing CV (Fig. 4a and Movie S2). We also measure the synchronization efficiency for four groups of large droplets with different CVs (Fig. S7b). Similarly, the synchronization efficiency of our device is above 98% and significantly higher than that of the negative device throughout the tests (Fig. 4b and Movie S3). It is worth mentioning that the highest value of CV in our experiment is set at 9%, although higher values of CV can also be tuned and synchronized. Droplets with CV values higher than 10% are regarded with non-uniform size, which is beyond the scope of this work^27^.

In addition, we also assess the utility of our design in balancing the fluctuations in reinjection flow rates. The percentage of one-to-one droplet pairing is measured at periodically varied flow rates with CVs ranging from 2 to 8% (Movie S4 and S5). For both the small and large droplets, the synchronization ratio of our device can remain high (over 98%) despite the fluctuations in flow rates. In contrast, the negative device fails to accommodate the fluctuations and shows significantly lower synchronization efficiencies (Fig. 4c, d).

### Regulation of the droplet pairing ratio

**Fig. 5:**
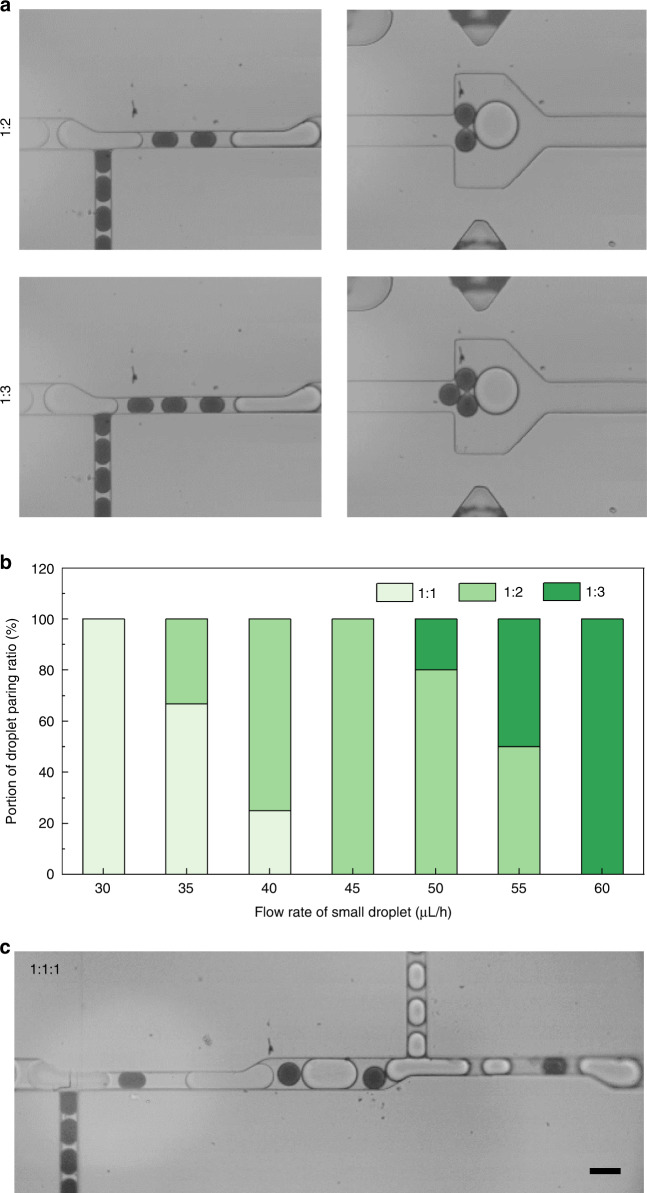
On-demand regulation of the droplet pairing ratio. **a** Micrographs showing the droplet arrangement and merging at the pairing ratios of 1:2 and 1:3. **b** Stacked bar chart showing the portion of droplets with different pairing ratios at different small-droplet flow rates. **c** Micrograph showing the arrangement of three sets of droplets at a pairing ratio of 1:1:1. Scale bar: 50 μm

Merging the droplets at a one-to-one pairing ratio can meet the requirements of a number of applications. Nevertheless, for some complex assays, such as the encapsulation of a controlled number of cells, higher ratios of droplet pairing and merging are frequently needed^28^. Using our device, the pairing ratio of large and small droplets can be regulated from 1:1 to 1:3 on demand by simply adjusting the reinjection flow rates (Fig. 5a and Movie S6). The flow rate of the large droplet is set constant at 100 μL/h, while the flow rate of the small droplet is adjusted from 30 to 60 μL/h. Initially, at a flow rate of 30 μL/h, all the droplets form a stable 1:1 pairing; as the flow rate increases, the portion of 1:2 droplet pairs gradually rises and reaches 100% at a flow rate of 45 μL/h; when the flow rate further increases, 1:3 droplet pairs are formed, and stable pairing is achieved at a flow rate of 60 μL/h (Fig. 5b). Hence, higher ratios of droplet pairing can be formed by further increasing the small-droplet flow rate.

In addition, in some scenarios where two types of samples are added, merging of three sets of droplets is needed^29^. To achieve this, we add one more injection channel in parallel to the former channel and reinject an additional set of small droplets without fluorescent dyes. By adjusting the flow rates of the large droplet, the first small droplet, and the second small droplet at 100, 30, and 30 μL/h, respectively, the three sets of droplets can be synchronized to form 1:1:1 pairs. First, the large droplet and the first small droplet are alternately arranged at the first junction; then, the second small droplet is inserted between the first small droplet and the next large droplet at the second junction; subsequently, the trains of the three droplets are spaced apart and merged in the downstream channel (Fig. 5c and Movie S7). In this manner, more parallel channels can be introduced to increase the number of droplets in each train.

### Multi-step reaction through droplet merging

**Fig. 6:**
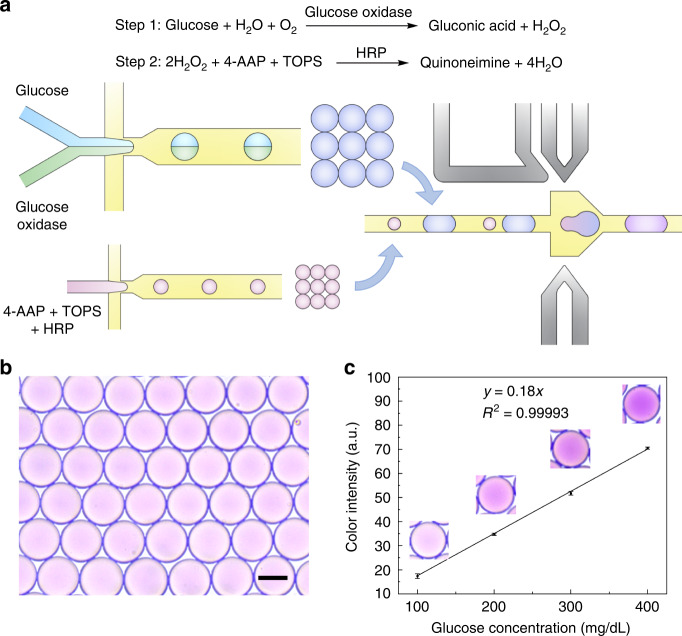
Multi-step reactions through droplet merging. **a** Schematic showing the process of droplet merging and the enzymatic reactions within the droplets. **b** Optical micrograph showing the merged droplets with uniform size and homogeneous quinoneimine. Scale bar: 50 μm. **c** Graph showing the variation in droplet color intensity with glucose concentration. Two hundred droplets were captured and analyzed at each concentration

High-efficiency droplet pairing and merging allow stable addition of reagents across multiple rounds of droplet processing^30,31^. To demonstrate the potential for high-throughput multi-step assays, we conducte a two-step enzymatic reaction within the droplets (Fig. 6a). In this reaction, glucose should be first oxidized by glucose oxidase so that excessive amounts of H_2_O_2_ are produced; then, 4-aminoantipyrine (4-AAP) and 3-(N-ethyl-3-methylanilino)propanesulfonic (TOPS) can be steadily oxidized by horseradish peroxidase (HRP) to produce quinoneimine, a compound with a violet color. Thus, we first coencapsulate glucose and glucose oxidase into large droplets. After 30 min of incubation, a sufficient amount of H_2_O_2_ is produced through oxidation of glucose by glucose oxidase. Then, the large droplets are merged with the small droplets containing 4-AAP, TOPS, and HRP to produce quinoneimine dyes in a stable manner (Movie S8). Upon completion of the cascade reaction, droplets with uniform size and color distribution are obtained, indicating the steady and homogeneous synthesis of quinoneimine within the droplets (Fig. 6b). Moreover, the amount of quinoneimine generated can be readily tuned by adjusting the glucose concentration. The color intensity of the resultant droplets, which is proportional to the quantity of quinoneimine, shows a linear relation with the glucose concentration, further confirming the stability and controllability of the droplet-based cascade reaction. The above results demonstrate that our system offers an ideal platform for precisely controlled multi-step reactions (Fig. 6c).

### Cell-bead pairing through droplet sorting and merging

**Fig. 7:**
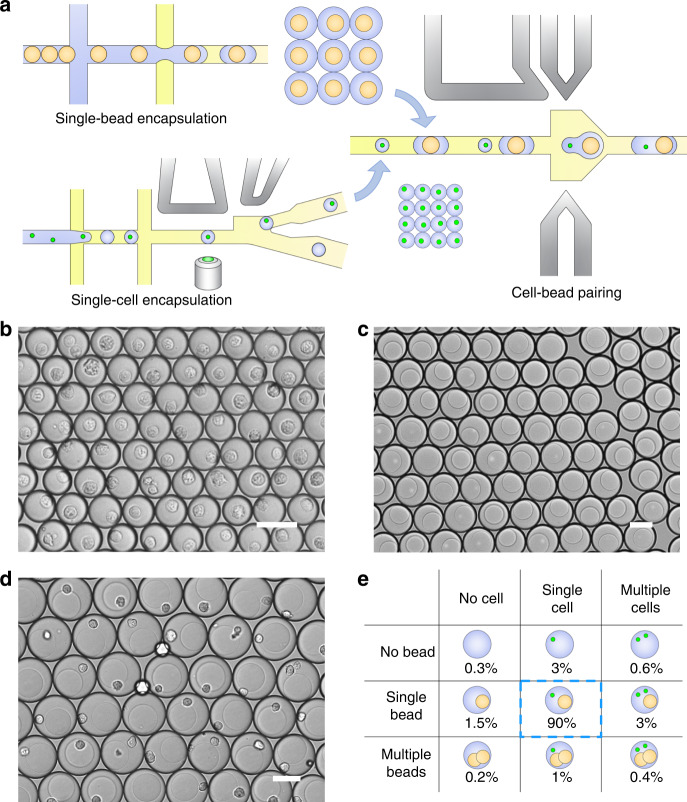
Cell-bead pairing through droplet merging. **a** Schematic showing the process of droplet manipulations for the pairing of single cells and single beads. Optical micrographs showing, **b** postsorting droplets encapsulating single cells, **c** droplets encapsulating single beads, and **d** postmerging droplets with cell-bead pairing. Scale bars: 50 μm. **e** Table depicting the proportion of droplets with different pairings of cells and beads

Our droplet merging system allows high-efficiency pairing of different types of discrete targets after rounds of droplet manipulations. For instance, single cells and single barcoding beads can be paired for applications, such as high-performance single-cell sequencing^32^. In conventional assays, although hydrogel beads can be closely packed and encapsulated in a controlled manner, the cell encapsulation is randomly distributed according to Poisson statistics^33^. As a result, less than 10% of the droplets can encapsulate both the target single cells and single beads, causing a significant loss of samples and reagents. To increase the encapsulation rate, we generate droplets with single cells and single beads separately, followed by subsequent merging in pairs (Fig. 7a). Droplets with single cells are first generated and sorted in an integrated device. The cell concentration is regulated to ensure that most droplets are empty and that only 10% contain single cells. Through sorting, over 95% of the droplets can encapsulate single cells, as shown in Fig. 7b. Subsequently, droplets with single acrylamide beads are generated. The hydrogel beads are closely packed and reinjected into the channel, thus beating the Poisson statistics to achieve a single-bead encapsulation rate of over 95% (Fig. 7c). Afterward, the two sets of droplets are reinjected into our merging device for cell-bead pairing (Movie S9). Ultimately, approximately 90% of the droplets encapsulate single cells and single beads, which is 9 times higher than conventional assays (Fig. 7d, e). The highly controlled pairing can significantly increase the droplet barcoding efficiency, thus reducing the cost of reagents and barcoding beads.

## Conclusions

In this study, we present a hydrodynamic resistance-regulated microfluidic design for self-synchronization of reinjected droplets. Each droplet alternately enters the junction and increases the local hydrodynamic resistance, thus transiently stopping the flow of another droplet and leading to stable droplet pairing and merging. Compared to conventional designs without enhancement of blockage, our design can achieve a synchronization efficiency of 100% even at a frequency mismatching rate of 10%. Moreover, high synchronization efficiencies (over 98%) can be achieved at nonuniform droplet size distributions and flow rate fluctuations. In addition, we successfully synchronize and merge different sets of droplets at tunable pairing ratios, including 1:1, 1:2, 1:3, and 1:1:1. To demonstrate the potential of our system to add reagents after rounds of droplet processing, we conduct a two-step enzymatic reaction by merging two groups of droplets containing enzyme/substrate. Moreover, using this system, we coencapsulate single cells and single hydrogel beads into droplets, paving the way for high-efficiency single-cell barcoding and sequencing. Likewise, this technique can be applied to pair two single cells for cell fusion and cell‒cell interaction studies^34^. Therefore, we believe our technique can enhance various droplet-based chemical assays and biological applications.

## Materials and methods

### Sample preparation

Fluorinated oil (HFE7500, 3 M, USA) supplemented with 0.5% (w/w) surfactant (RAN Biotechnologies, USA) was used as the continuous phase. To visually differentiate the two sets of droplets, deionized (DI) water was used as the first dispersed phase to generate large droplets, while 12 mM fluorescent dye solution (Rhodamine 6 G, Sigma‒Aldrich, USA) was used as the second dispersed phase to generate small droplets. The interfacial tension is 6.8 mN/m. During droplet synchronization, the interfacial tension forces help resist changes in droplet shape, preventing droplet breakup.

For enzymatic reactions, glucose oxidase (G7141), D-(+)-glucose (G7021), 4-AAP (A4382), and TOPS (E8506) were all purchased from Sigma‒Aldrich, and HRP (31490) was purchased from Thermo Scientific. The concentrations of glucose oxidase, HRP, 4-AAP, and TOPS were maintained at 200 U/mL, 1200 U/mL, 0.6 mol/L, and 1 mol/L, respectively, while the concentration of glucose was set at 100, 200, 300, and 400 mg/dL.

Mouse myeloma cells (SP2/0 cell line) were used as the model cells for cell-bead pairing. They were cultured in Roswell Park Memorial Institute (RPMI) 1640 Medium (Life Technologies, USA) supplemented with 10% fetal bovine serum (FBS, A4766801, Gibco, USA) and 100 U/mL penicillin/streptomycin (Gibco, USA) in a humidified atmosphere with 5% CO_2_ at 37 °C. After trypsin treatment, the cells were resuspended in 2 μM Calcein AM (C3100MP, Invitrogen, USA) in FBS-free cell culture medium for 30 min to enable live cell staining. Then, the cells were centrifuged, and 15% (v/v) OptiPrep (D1556, Sigma‒Aldrich, USA) in cell culture medium, which had the same density as the SP2/0 cells, was used for resuspension of the cells.

### Device fabrication

The microfluidic device was fabricated using a typical soft lithography replica molding technique^35^. First, a channel mold with a height of 40 μm was fabricated on a silicon wafer (N100, University, USA) with SU-8 photoresist (2025, MicroChem, USA) using maskless lithography (SF-100 Xcel, Intelligent Micro Patterning, LLC, USA). Then, PDMS prepolymer base (Sylgard 184, Dow Corning, USA) was mixed with curing agent at a weight ratio of 10:1 by a conditioning mixer (AR-100, THINKY, JP). The mixture was poured onto the channel mold and cured at 65 °C for 4 h. Subsequently, the PDMS layer was peeled off from the mold, followed by bonding to a glass substrate through oxygen plasma treatment (PDC-002, Harrick, USA) and heating at 90 °C for 2 h. To fabricate the electrodes for droplet coalescence, empty channels were fabricated in the desired shapes on both sides of the main channel. A low-melting-point metal wire (52225, Indium Incorporation, USA) was then inserted into one end of the channel at 95 °C, and negative pressure was applied to another end to fill the whole channel with liquid metal. After further cooling for solidification, the electrodes were formed into desired shapes. The whole channel was treated with aquapel (PPG, USA) to guarantee stable droplet generation and manipulations.

### System setup

Uniformly sized droplets were generated using high-precision syringe pumps (neMESYS 290 N, CETONI, GER) to control the flow rates. The generated droplets were reinjected into the merging device after most of the carrier oil was removed from the emulsions. As a result, the droplets were closely packed and flowed into the channel with uniform gaps. A signal generator (DG1022U, RIGOL Technologies, CN) and a high-voltage amplifier (5/80, Trek, USA) were used to supply the device with a 30 kHz alternating current (AC) signal for droplet coalescence. A high-speed camera (Phantom V9.1, Vision Research, USA) was used to capture the images and record the videos.

To generate droplet sizes with varied CV, we mixed three sets of droplets with different diameters at a 1:1:1 ratio (Table 2). When the small-droplet diameter was varied, the large-droplet diameter was kept constant at 65 μm; when the large-droplet diameter was varied, the small-droplet diameter was kept constant at 35 μm. To create fluctuated flow conditions, we changed the reinjection flow rates among three values periodically with each flow rate lasting for 5 s (Table 3). When the small-droplet flow rate was varied, the large-droplet flow rate was set constant at 100 μL/h; when the large-droplet flow rate was varied, the small-droplet flow rate was set constant at 30 μL/h.

**Table 2:** Sizes of small and large droplets for the CV of 3, 5, 7, and 9% and the tested flow rates

CV	Droplet size	Small-large droplet flow rates
3% (small)	33.5 μm–35 μm–36.5 μm	20 μL/h–85 μL/h
5% (small)	35 μm–37.5 μm–40 μm	20 μL/h–60 μL/h
7% (small)	36.5 μm–40 μm–43.5 μm	20 μL/h–65 μL/h
9% (small)	31 μm–35 μm–39 μm	20 μL/h–80 μL/h
3% (large)	62.5 μm–65 μm–67.5 μm	20 μL/h–80 μL/h
5% (large)	58 μm–62 μm–66 μm	20 μL/h–80 μL/h
7% (large)	58.5 μm–64 μm–69.5 μm	20 μL/h–70 μL/h
9% (large)	58 μm–65 μm–72 μm	20 μL/h–75 μL/h

**Table 3:** Flow rates of small and large droplets for the CV of 2, 4, 6, and 8%

CV	Flow rate 1	Flow rate 2	Flow rate 3
2% (small)	29.4 μL/h	30 μL/h	30.6 μL/h
4% (small)	28.8 μL/h	30 μL/h	31.2 μL/h
6% (small)	28.2 μL/h	30 μL/h	31.8 μL/h
8% (small)	27.6 μL/h	30 μL/h	32.4 μL/h
2% (large)	98 μL/h	100 μL/h	102 μL/h
4% (large)	96 μL/h	100 μL/h	104 μL/h
6% (large)	94 μL/h	100 μL/h	106 μL/h
8% (large)	92 μL/h	100 μL/h	108 μL/h

### Cell-bead pairing

A cell density of 4.5 × 10^6^ cells/mL was used so that approximately 10% of the generated droplets contained single cells. The cell sorting system was set up as described in previous works^36^. The flow rates of the cell solution, generation oil, and spacing oil were set at 200, 800, and 2000 μL/h, respectively, resulting in stable generation and sorting of single-cell encapsulation droplets at a frequency of 1 kHz. Throughout a 30-min operation, approximately 0.2 million droplets with single cells are harvested.

The polyacrylamide hydrogel beads were fabricated by polymerizing microfluidic droplets^37^. The dispersed phase included 10 mM Tris-HCl, 1 mM EDTA, 15 mM NaCl, 6.2% acrylamide, 0.2% bis-acrylamide, and 0.3% ammonium persulfate. The continuous phase was HFE7500 containing 0.4% TEMED and 2% surfactant. Droplets with a diameter of 34 μm were generated at a frequency of 1.5 kHz, followed by incubation at 65 °C for 12 h to induce polymerization. Then the formed hydrogel beads were extracted by adding demulsifier, 1H,1H,2H,2H-Perfluorooctanol (97%, Thermo Scientific, USA), to destabilize the surfactant and break the droplets, followed by removing the carrier oil and washing with phosphate-buffered saline (PBS). After centrifugation and removal of PBS, the fabricated beads were closely packed and reinjected into the microfluidic channel to generate bead-encapsulating droplets. The flow rates of the bead solution, spacing cell culture medium, and oil were set at 300, 400, and 500 μL/h, respectively, resulting in stable generation of droplets encapsulating a single bead at a frequency of 1.7 kHz. Throughout a 5-min operation, approximately 0.5 million droplets with single beads are generated. Finally, the two sets of droplets encapsulating single cells and single beads were reinjected into the merging device for one-to-one pairing.

### Statistical analysis

The synchronization ratio is measured by recording 200 events of droplet pairing and calculating the percentage of one-to-one pairing. The recording is repeated every 5 min within a duration of 20 min to obtain the average and standard deviation of synchronization ratio, which can well reflect the robustness of this system. Student’s t tests were performed to compare the percentage of one-to-one pairing at nonuniform droplet sizes and fluctuating flow rates between our device and the negative-control device. A difference was regarded as significant when *p* < 0.05, and the statistically significant difference was expressed as “*”.

## Supplementary information

supplemental material

Movie. S1

Movie. S2

Movie. S3

Movie. S4

Movie. S5

Movie. S6

Movie. S7

Movie. S8

Movie. S9
