# Supplementary material for: Self-synchronization of reinjected droplets for high-efficiency droplet pairing and merging
Source: Microsyst Nanoeng. 2023 Mar 9;9:24. doi: 10.1038/s41378-023-00502-6 (PMC9995457; doi:10.1038/s41378-023-00502-6)
Supplement: Supplementary file 1 — supplemental material [file 41378_2023_502_MOESM1_ESM.docx]

**Supplementary material**


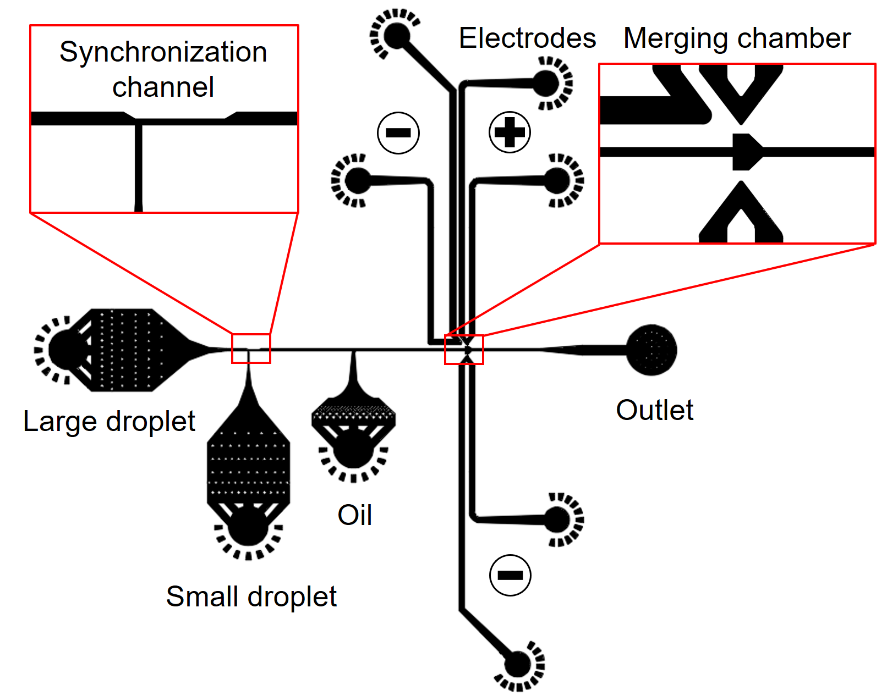


Fig. S1 Design of the microfluidic channel.


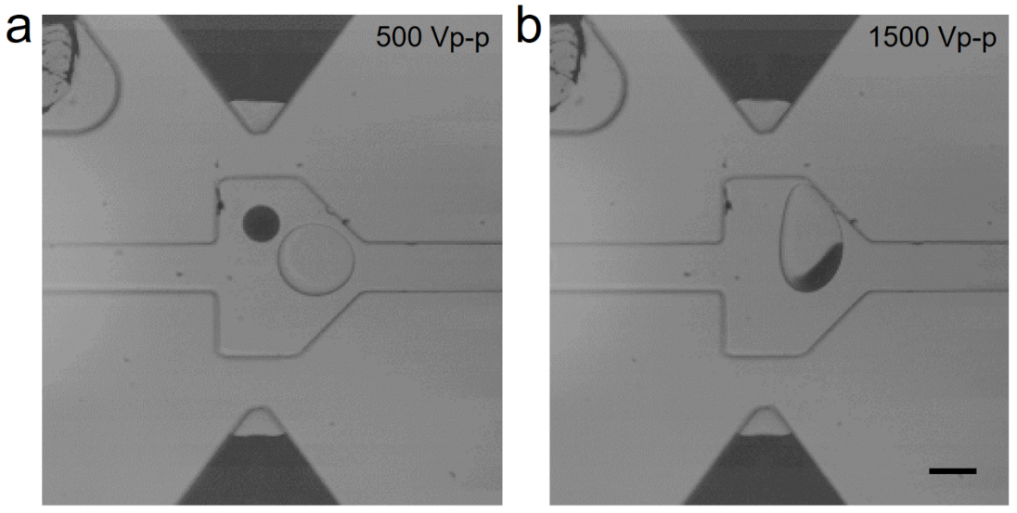


Fig. S2 Micrographs of (a) non-merged droplets under the voltage of 500 Vp-p and (b) sidewall-wetting droplets under the voltage of 1500 Vp-p. Scale bar: 50 μm.


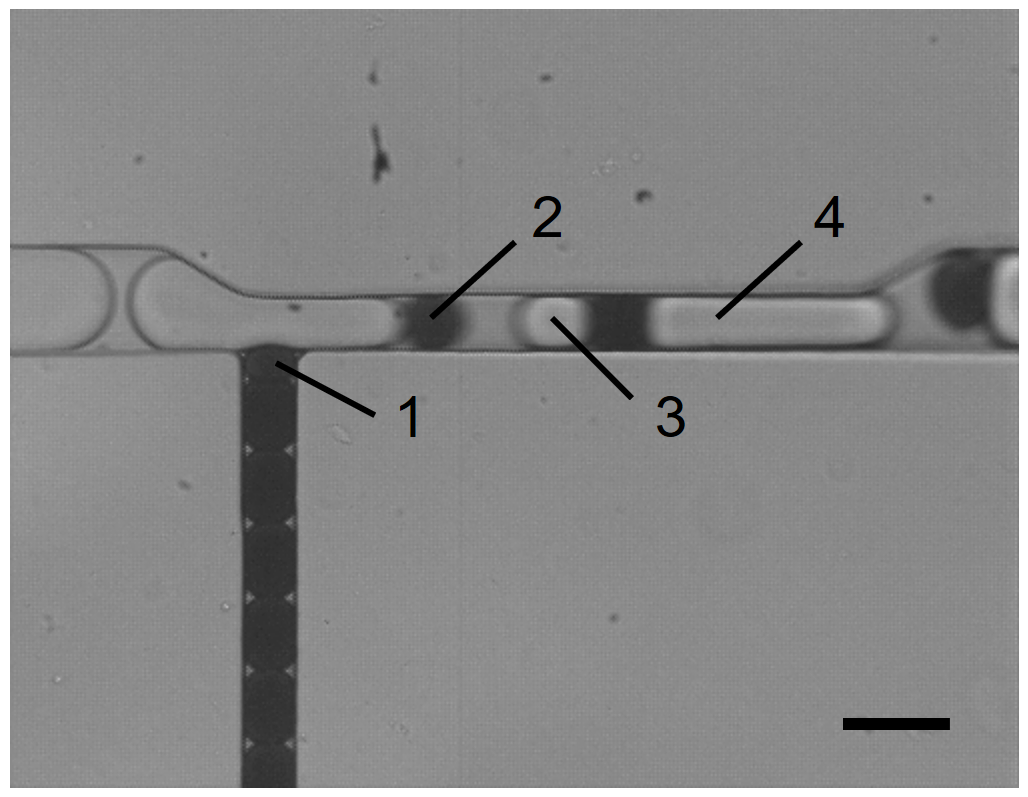


Fig. S3 Micrographs showing the droplet breakup under the flow rates of 80 μL/h and 400 μL/h. The droplets 1 and 2 are originated from the same small droplet that is split in the junction, while the droplets 3 and 4 are originated from the same large droplet. Scale bar: 50 μm.


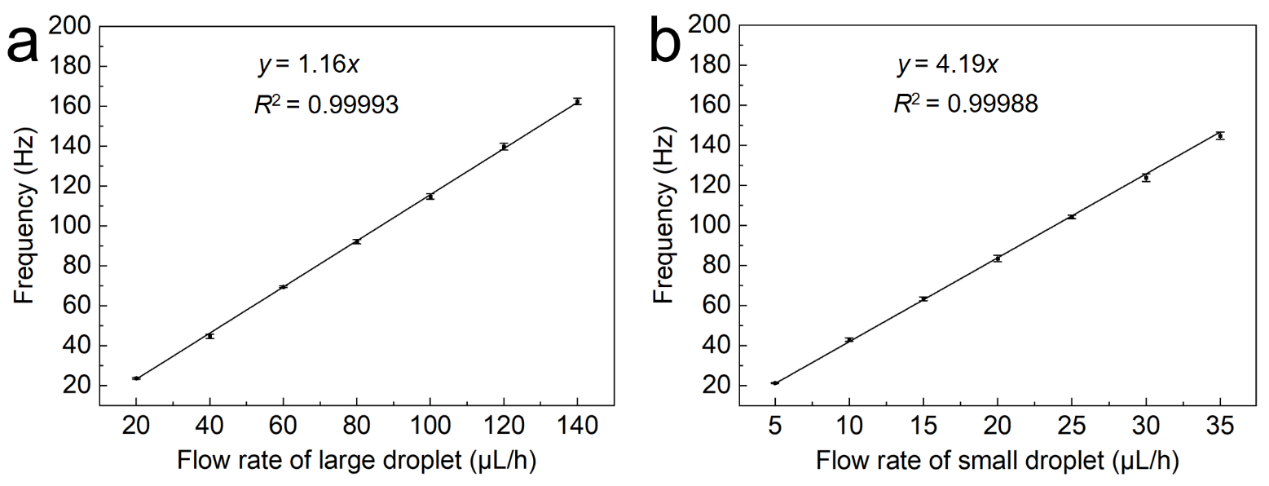


Fig. S4 Graphs showing the linear relationship between the frequency and the flow rate for (a) the large droplets and (b) the small droplets.


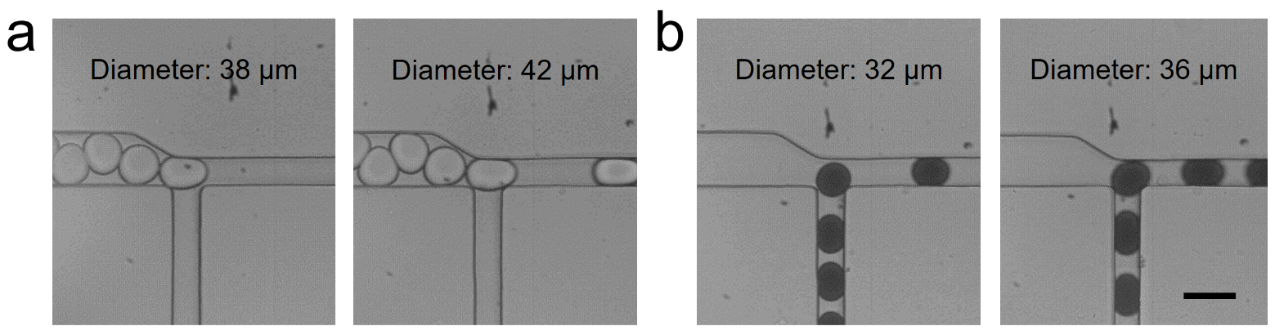


Fig. S5 Micrographs showing the channel blockage at different droplet sizes. (a) The large droplet with the diameter of 38 μm cannot block the junction, while the 42-μm droplet presents the blockage effect. (b) The small droplet with the diameter of 32 μm cannot block the junction while the 36-μm droplet present the blockage effect. Scale bar: 50 μm.


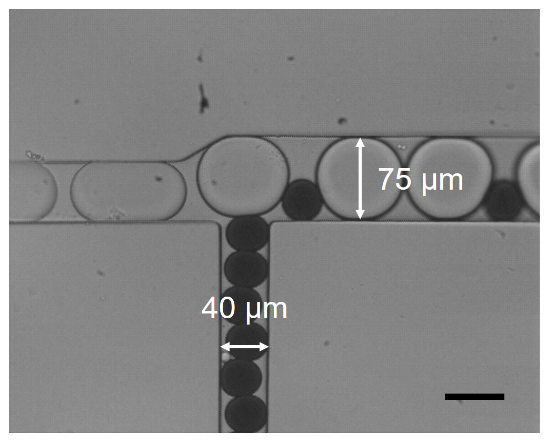


Fig. S6 Micrographs showing the channel geometry of the negative-control device. The droplets fail to block each other at the junction with a dimension of 40 μm and 75 μm. Scale bar: 50 μm.


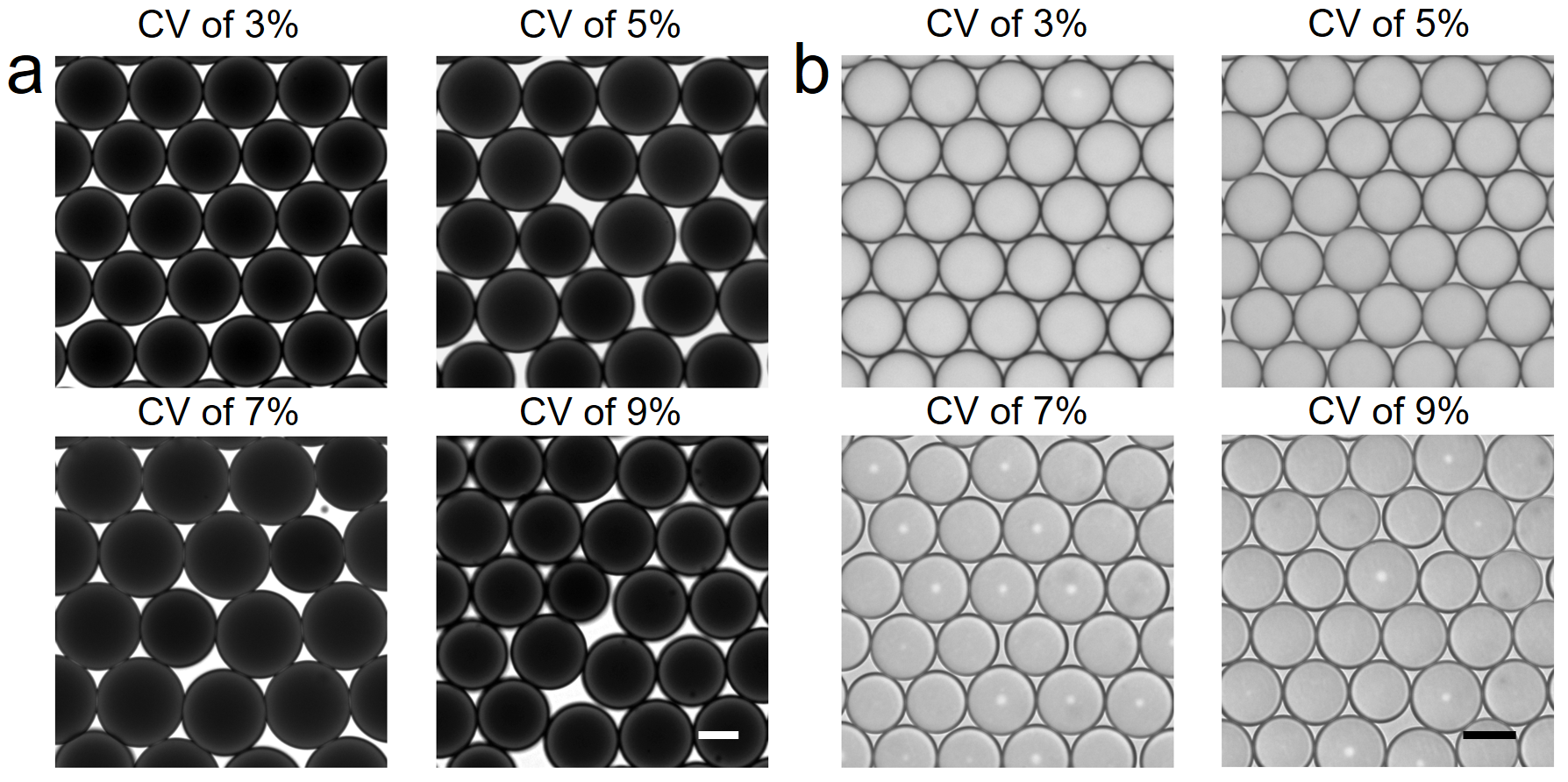


Fig. S7 Micrographs showing the morphology of (a) the small droplets and (b) the large droplets with varied CVs of the diameter. Scale bar in (a): 20 μm; scale bar in (b): 50 μm.

Movie. S1 Synchronization, spacing, and merging of reinjected droplets. The movie is recorded at 5000 fps and played at 30 fps.

Movie. S2 Synchronization of small droplets with varied size. The movie is recorded at 2000 fps and played at 20 fps.

Movie. S3 Synchronization of large droplets with varied size. The movie is recorded at 2000 fps and played at 20 fps.

Movie. S4 Synchronization of small droplets with fluctuated flow rate. The movie is recorded at 2000 fps and played at 20 fps.

Movie. S5 Synchronization of large droplets with fluctuated flow rate. The movie is recorded at 2000 fps and played at 20 fps.

Movie. S6 Synchronization and merging of droplets with different pairing ratios. The movie is recorded at 2000 fps and played at 10 fps.

Movie. S7 Synchronization and merging of three sets of droplets. The movie is recorded at 1000 fps and played at 10 fps.

Movie. S8 Droplet merging for the enzymatic cascade reaction. The movie is recorded at 1000 fps and played at 10 fps.

Movie. S9 Droplet merging for cell-bead pairing. The movie is recorded at 1000 fps and played at 10 fps.
